# Acute fulminant necrotizing amoebic colitis: a rare and fatal complication of amoebiasis: a case report

**DOI:** 10.4076/1757-1626-2-6557

**Published:** 2009-09-11

**Authors:** Shilpi Singh Gupta, Onkar Singh, Sumit Shukla, Mathur K Raj

**Affiliations:** Department of Surgery, M.G.M Medical College & M.Y. HospitalIndore, 452001India

## Abstract

Acute Fulminant Necrotizing Amoebic Colitis is a rare complication of amoebiasis that is associated with high mortality. Only one to four such cases are seen per year in large hospitals of India, and only few such cases have been reported in the literature. The condition requires early diagnosis and surgical intervention. We recently cared for a patient who presented with acute abdomen with history of intermittent abdominal pain and diarrhea. Before presenting to our institution he was misdiagnosed as a case of inflammatory bowel disease and had been treated with steroids. On emergency exploration, extensive necrosis and multiple perforations in retroperitoneum involving entire colon were seen. Total colectomy with ileostomy was performed. Postoperative course was marked by septicaemia and multi-organ failure followed by death. This case report emphasizes the importance of early diagnosis and treatment of acute FAC, and associated high mortality.

## Introduction

Amoebiasis is a parasitic infection common in under-developed countries and among populations with low socio-economic status living in congested localities with poor sanitation. Causative organism is a protozoon; *Entamoeba histolytica*, that principally affects colon and liver. Around 100,000 people die each year worldwide from amoebic colitis and amoebic liver abscess. The majority of infested humans with intestinal illness remain asymptomatic. However, very uncommonly the disease takes a fulminant super-acute course because of development of Necrotizing Amoebic Colitis, which has very high mortality ranging from 55% to 100% if diagnosis and treatment is delayed. Diagnosis is difficult and frequently confused with inflammatory bowel disease leading to wrong treatment with steroids with devastating results. Perforation is common in FAC, and peritonitis is the commonest cause of death. Primary total resection of involved segment and exteriorization of the proximal and distal transected ends, and bowel reconstruction 3-6 months later, is the treatment of choice. Also, the literature favors early presumptive antiamoebic treatment in cases of severe and undiagnosed colitis in endemic areas.

## Case presentation

We present a case of 68-years-old Indian, Hindu male patient who landed in emergency room with severe pain over lower abdomen associated with distention of abdomen and bloody diarrhea of one day duration. Patient gave history of intermittent lower abdominal pain and diarrhea for last eight months. Before coming to our institute he was diagnosed as a case of inflammatory bowel disease and was being treated with acetyl-salisylic acid (Mesacol 800 mg TDS) and intermittent steroid therapy for eight months. He was a chronic alcoholic and smoker. On examination, patient looked toxic with pulse rate of 126/minute. Tenderness and guarding was present all over abdomen. Blood samples were immediately sent for routine investigations, which were significant for the presence of leucocytosis (18,300/cmm), Na^+^ 129 mEq/l and K^+^ 2.9 mEq/l. ELISA test for HIV detection was negative. Urine sample was also sent for routine microscopy which showed few pus cells and presence of E. coli. Abdominal radiograph in erect position showed dilated bowel loops but no free air under the diaphragm. Ultrasonography of abdomen was asked for, which revealed distended large bowel loops with no fluid collection in peritoneal cavity. On per rectal examination rectum was empty and examining finger was tinged with blood and mucous.

**Figure 1.**
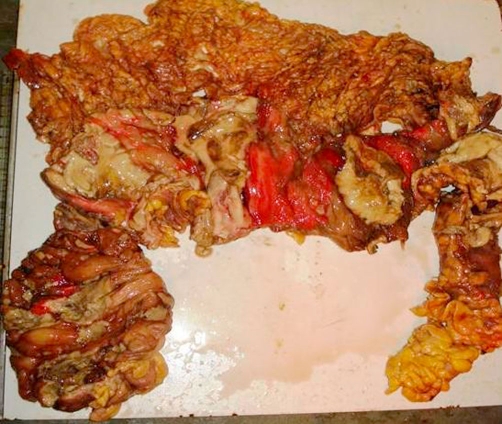
Total colectomy specimen (Opened), showing multiple large geographic ulcers covered with necrotic purulent slough.

**Figure 2.**
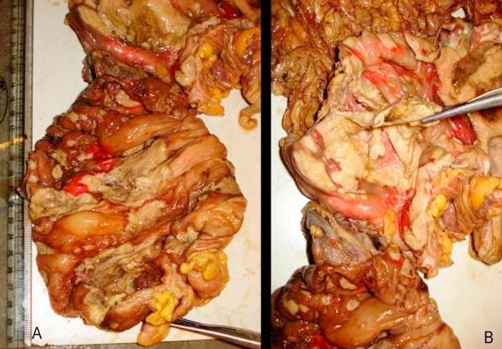
**(A)** Ceacal area; and **(B).** Hepatic flexure of resected colon, showing large ulcers covered with plaques of yellowish necrotic material, appendices is pointed by forceps.

**Figure 3.**
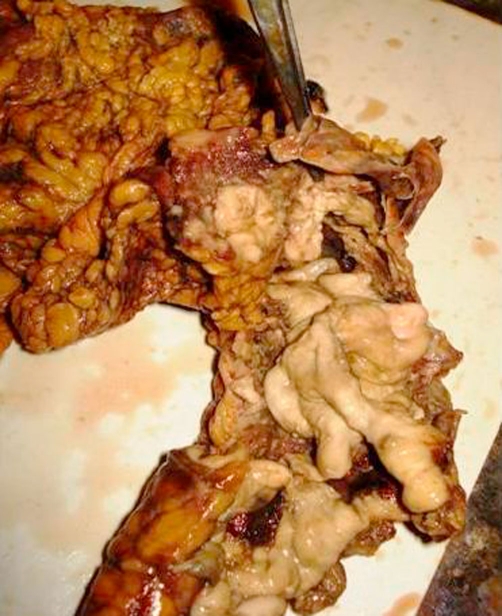
Splenic flexure of resected colon; large necrotic area with perforation has been shown.

**Figure 4.**
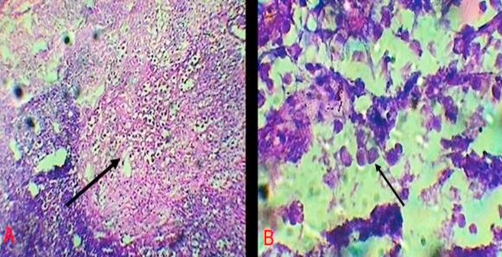
**(A)** H & E stained tissue from ulcer base from the resected specimen of colon; arrow indicates the area full of colonies of trophozoits of *E. histolytica*. (10× magnification), **(B)** Same area under high power (40× magnification); a colony of trophozoits of *E. histolytica* can be clearly seen, indicated by arrow.

Intravenous antibiotics in the form of a combination of ceftriaxone, amikacine and metrogyl were started and patient was taken for exploratory laparotomy. Intra-operatively, multiple large areas of necrosis were found involving almost entire colon. Almost whole length of ascending and descending colon, and proximal sigmoid colon was perforated into the retro-peritoneum at multiple sites. Pelvic cavity was free from any fluid collection but minimal fluid was found in retroperitoneal space. Total colectomy with proximal ileostomy and exteriorization of distal end of sigmoid colon as mucous fistula was done. Luminal surface of resected colon had multiple ulcers measuring 3 mm to 5 cm, covered at place with necrotic purulent slough (Figure 1, 2, 3). Diagnosis of amoebic colitis was confirmed postoperatively on histo-pathological examination which showed necrotizing trans-mural ulceration with inflammatory exudates, and trophozoites of *Entamoeba histolytica* as isolated and in colonies at the ulcer bases (Figure 4A & B). Sections from the appendics showed features of nonspecific appendicitis. Terminal 6 cm portion of ileum was essentially normal. Postoperative course was complicated by wound infection and septicaemia. Unfortunately we lost our patient on 12^th^ postop day due to multiple organ failure.

## Discussion:

*Entamoeba histolytica;* the causative organism of amoebiasis, is a protozoan parasite which affects two main organ systems in human body: Gastrointestinal tract and the liver. Gastrointestinal involvement occurs as a result of ingestion of the cysts of the parasite from food or water contaminated with faeces. The cysts are digested in the intestinal lumen releasing trophozoites. The trophozoites reproduce by clonal expansion and subsequently form cysts which are excreted in the faeces to start a new cycle [1].

Amoebiasis may involve any part of the bowel, but it has a predilection for the cecum and ascending colon [2]. Presentation of the intestinal illnesses has a spectrum ranging from aymptomatic infection, symptomatic noninvasive infection, acute protocolitis (dysentery) to fulminant colitis with perforation [3]. The majority (90%) of humans harbouring Entamoeba histolytica, fall into the group of asymptomatic carriers and live normal life [1,4]. Only in 6%-11% of patients with symptomatic infection [5], the most virulent host response to the amoebic infection occurs leading to fulminating reaction, that leads to necrotizing colitis and perforation, peritonitis, and death [4]. Such course of amoebiasis in the form of acute fulminant necrotizing amoebic colitis (FNAC) is rare and only a few such cases of have been reported in the literature [6].

Development of such fulminant course is found to be associated with various factors including male gender, age over 60 years, associated liver abscess, progressive abdominal pain, and signs of peritonitis, leukocytosis, hyponatremia, hypokalemia, and hypoalbuminemia [7]. FAC, in majority of cases has characteristic symptoms and signs such as severe abdominal distention and pain with peritoneal signs, sepsis with high fever, watery or bloody mucoid diarrhea and dehydration [8,9]. Peritonitis develops either because of frank perforation or a slow leak through an extensively diseased bowel [4,10].

Apart from its rarity, clinical significance of fulminant amoebiasis lies in the fact that the condition is difficult to diagnose and treat, and associated with a very high mortality rate [11]. Diagnosis is often confused with idiopathic inflammatory bowel disease resulting in erroneous administration of steroids. Colonoscopic appearance and colonic tissue biopsy are helpful in differentiating amoebiasis from other forms of colitis. Clinical symptoms, laboratory studies and X-ray findings are insufficient to make an accurate diagnosis [12,13]. Conventional method of microscopic examination of stool is less sensitive (25% to 60%). Antigen detection both in the patient’s stool and serum is more sensitive and specific method [14,15]. Pathology of the invaded colonic tissue shows transmural inflammation widespread necrosis along with large numbers of amoebic trophozoites within the inflammatory exudates [16].

For acute amoebic colitis, once suspected, early diagnosis and aggressive supportive and antiamoebic treatment should be instituted. If fulminant colitis develops, the outcome is poor with mortality ranging from 55% to 87.5% [17], peritonitis being the commonest cause of death [18]. Early diagnosis and surgical treatment significantly decrease mortality [8,9]. It has been stated that conservative surgery has no place in the management of acute FAC, and primary total resection is the treatment of choice [19]. Because there is a high risk of suture breakdown in tissue containing amoebae; a staged operation in the form of exteriorization of the proximal and distal transected ends, and bowel reconstruction 3-6 months later, is highly recommended for FNAC [20]. Also, in endemic areas, patients with severe and undiagnosed colitis should be treated presumptively with specific antiamoebic therapy until a diagnosis of amoebic colitis can be excluded [8,9,19].

## Conclusion

The key message is to emphasize the possibility of acute necrotizing FAC as a rare complication of amoebiasis, and poor outcome associated with misdiagnosis and use of steroids. Early recognition and antiamoebic treatment, along with urgent aggressive resectional surgery with exteriorization of the proximal and distal bowel ends, are needed to reduce mortality from acute fulminant necrotizing amoebic colitis.

## Abbreviations

FAC: fulminant amoebic colitis
FNAC: fulminant necrotizing amoebic colitis
